# Contact among healthcare workers in the hospital setting: developing the evidence base for innovative approaches to infection control

**DOI:** 10.1186/s12879-018-3093-x

**Published:** 2018-04-17

**Authors:** Krista M. English, Joanne M. Langley, Allison McGeer, Nathaniel Hupert, Raymond Tellier, Bonnie Henry, Scott A. Halperin, Lynn Johnston, Babak Pourbohloul

**Affiliations:** 1 Institute for Resources, Environment and Sustainability, University of British Columbia, 2202 Main Mall, Vancouver, BC V6T 1Z4 Canada; 2 Departments of Pediatrics, and Community Health & Epidemiology, Canadian Center for Vaccinology, IWK Health Centre, Nova Scotia Health Authority, Dalhousie University, Halifax, NS B3K 6R8 Canada; 3 Mount Sinai Hospital, 600 University Avenue, Toronto, ON M5G 1X5 Canada; 4 Weill Cornell Medicine, 402 East 67 St, New York, NY 10065 USA; 5 Department of Pathology & Laboratory Medicine, And Provincial Laboratory for Public Health of Alberta, 3030 Hospital Drive NW, Calgary, AB T2N 4W4 Canada; 6 British Columbia Ministry of Health, 1515 Blanshard St, Victoria, BC V8W 9P4 Canada; 7 Departments of Pediatrics, and Microbiology & Immunology, Canadian Center for Vaccinology, IWK Health Centre, Nova Scotia Health Authority, Dalhousie University, Halifax, NS B3K 6R8 Canada; 8 Department of Medicine, Dalhousie University & Nova Scotia Health Authority, Halifax, NS B3H 1V7 Canada

**Keywords:** Hospital associated infections, Infection prevention and control, Contact networks

## Abstract

**Background:**

Nosocomial, or healthcare-associated infections (HAI), exact a high medical and financial toll on patients, healthcare workers, caretakers, and the health system. Interpersonal contact patterns play a large role in infectious disease spread, but little is known about the relationship between health care workers’ (HCW) movements and contact patterns within a heath care facility and HAI. Quantitatively capturing these patterns will aid in understanding the dynamics of HAI and may lead to more targeted and effective control strategies in the hospital setting.

**Methods:**

Staff at 3 urban university-based tertiary care hospitals in Canada completed a detailed questionnaire on demographics, interpersonal contacts, in-hospital movement, and infection prevention and control practices. Staff were divided into categories of administrative/support, nurses, physicians, and “Other HCWs” - a fourth distinct category, which excludes physicians and nurses. Using quantitative network modeling tools, we constructed the resulting HCW “co-location network” to illustrate contacts among different occupations and with locations in hospital settings.

**Results:**

Among 3048 respondents (response rate 38%) an average of 3.79, 3.69 and 3.88 floors were visited by each HCW each week in the 3 hospitals, with a standard deviation of 2.63, 1.74 and 2.08, respectively. Physicians reported the highest rate of direct patient contacts (> 20 patients/day) but the lowest rate of contacts with other HCWs; nurses had the most extended (> 20 min) periods of direct patient contact. “Other HCWs” had the most direct daily contact with all other HCWs. Physicians also reported significantly more locations visited per week than nurses, other HCW, or administrators; nurses visited the fewest. Public spaces such as the cafeteria had the most staff visits per week, but the least mean hours spent per visit. Inpatient settings had significantly more HCW interactions per week than outpatient settings.

**Conclusions:**

HCW contact patterns and spatial movement demonstrate significant heterogeneity by occupation. Control strategies that address this diversity among health care workers may be more effective than “one-strategy-fits-all” HAI prevention and control programs.

**Electronic supplementary material:**

The online version of this article (10.1186/s12879-018-3093-x) contains supplementary material, which is available to authorized users.

## Background

Nosocomial, or healthcare-associated infections (HAI) are a major burden to public health and the functioning of modern healthcare systems. In Canada, more than 200,000 patients acquire a HAI annually, and as a result, an estimated 8000 die [1]. Figures in the United States and Europe are comparable on a per-capita basis [2, 3]. The 2003 severe acute respiratory syndrome (SARS) outbreaks, and more recently of Middle East respiratory syndrome (MERS), highlight the major threat posed by HAIs, both within the hospital and for the wider community. Close contact between patients and/or healthcare workers (HCWs), and high concentrations of medically-vulnerable populations, combined with physical movement between treatment areas, are factors that may facilitate HAI spread within health care institutions and the community.

Current infection prevention and control (IPC) measures focus on proper performance of both routine practices (e.g. hand and respiratory hygiene) and additional precautions (e.g. airborne, contact and droplet precautions) by all HCWs [4, 5]. Before patient contact, HCWs determine precautions to be taken based on their own situational risk assessment. However, heterogeneity of collective contacts among patients and HCWs are not specifically addressed in the current guidelines. Preliminary attempts to quantify mixing patterns and contact rates have been conducted among the general population on a large scale [6–9], or in non-healthcare settings [10–12], but rates of HCW contacts within healthcare settings are postulated to be significantly higher and more heterogeneous than those within the general population [13].

Studies using electronic medical records to examine spatial movement throughout the hospital provide information on only a small subset of hospital interactions. These studies capture patient movement as it pertains explicitly to the more complex clinical services they receive but fail to capture HCW social or casual movement, such as visits to the cafeteria or meeting rooms, or some types of clinical contact (e.g. a second staff member assisting with patient mobilization or bathing, or cross-covering a colleague on break) [14–16]. Contact patterns for HCWs have been examined using radio frequency identification (RFID) tags, mote-based sensors, and direct observation [17–20]. These formats have suggested the potential for “super spreaders” in the hospital setting [20], and notable differences in contact patterns between occupations [19]. Since these studies are currently only within a single ward or unit, they are limited in generalizability to a hospital-wide setting since they do not take contacts outside the study setting into account. In addition to room-level contacts, it is important to note the patterns of movement throughout the hospital. This may reveal locations that can more readily propagate infection spread during outbreak scenarios.

Understanding the movement and contact patterns of HCWs within hospital settings may allow for more targeted and effective infection control interventions. To address this knowledge gap, we conducted a cross-sectional study of HCW in three major Canadian health care facilities to assess interpersonal contact patterns, movement throughout the facility, and demographic characteristics. These data can be used to develop a model that represents the heterogeneous contact patterns in the hospital setting. Additional questions on IPC practices were included to help parameterize future models of HAI reduction interventions.

## Methods

Using architectural maps and floor plans, site-specific surveys were created for three urban university-affiliated tertiary care Canadian hospitals (hereafter called Hospital A, B and C). The data collection instruments were hard-copy paper booklets with information packages, containing guidelines and rationale for the study, and 1 online survey. Employees were invited to participate through personal invitations, email and posters. Surveys were also attached to employee paystubs on two separate occasions. The paper surveys were to be completed by HCWs and returned anonymously to a centrally-located drop box. Local study staff informed participants that survey completion was voluntary and anonymous. This project was funded by the Canadian Institutes of Health Research (CIHR) and called the CONNECT I study. Ethics review boards at all participating universities and hospitals approved the project.

An estimated 8100 staff working (or volunteering) in any of the three hospitals were eligible to participate (~ 4100, ~ 2400, and ~ 1600 in Hospitals A, B and C, respectively). Our pre-survey target for participation was 1000 or 12.5%. The survey identified 19 different HCW occupational categories including attending and resident physicians, nurses, technicians, support staff, undergraduate trainees and other hospital workers who have patient contact. For this publication, all occupational categories other than physicians, nurses, and administrative/support staff are grouped together as a fourth main category called “other HCWs” (hereafter, oHCW). These categories are summarized in Table 1.

**Table 1:** Classification of aggregate categories based on self-reported occupations

	Self-reported Occupation	Category
1	Central Supply Technician	Admin/Support
2	Housekeeping	
3	Receptionist	
4	Service Assistant	
5	Volunteer	
6	Ward Clerk	
7	Nurse	Nurse
8	Nursing Student	
9	Staff Physician	Physician
10	Postgraduate Medical Trainee	
11	Medical trainee	
12	Medical Imaging Technologist	oHCW
13	Patient Attendants/Sitters	
14	Pharmacist	
15	Physiotherapist/Occupational Therapist	
16	Respiratory Therapist	
17	Social Worker	
18	Other^a^	
19	Other Student Discipline^a^	

^a^Respondents were assigned to one of the four above categories based on their description in the free-text field provided

The surveys collected demographics, spatial movement, and patient interaction (contact) data, as well as self-reported compliance with IPC practices by both the survey respondent and his or her coworkers. Direct patient contact was defined as two or more individuals coming within 1 m (approximately 3 ft) of each other for 2 min or more. This proximity has long been proposed as a guideline for the range of transmission of infection by large droplets. At the time of conducting the survey, this proximity and duration were estimated to be necessary but not sufficient for respiratory infection transmission (in more recent guidance, 2 m is considered the radius for potential transmission [21]). Indirect contact was defined as two or more individuals co-locating in the same room but not closer than 1 m.

For demographic analyses, differences between groups were assessed using Chi-square tests and analysis of variance (ANOVA).

Hospital floors were identified as predominantly patient-care area (PCA), predominantly non-patient-care area (non-PCA) and mixed (mPCA), by local study staff. Respondents reported the amount of time (in hours, or minutes) they averaged weekly in each location within their hospital. Detailed spatial locations such as pre-admission unit, day surgery unit, ambulatory internal medicine clinic or cafeteria, were identified in the questionnaire corresponding to each hospital. There were 251, 122, and 97 units in Hospitals A, B and C, respectively. The frequency of visits and mean reported hours were quantified for each location, and analysis of variance (ANOVA) was used to compare the groups. Tukey pairwise tests were used post-hoc to identify significant comparisons. Given the diversity and frequency of these small locations, it was necessary to aggregate the information that is simple to present and consistent across all sites. Since this paper concerns the structure of interpersonal HCW contacts and does not address the transmission dynamics of infection spread, we group these small locations to present the results for each actual hospital floor, as a spatial unit.

Infection prevention and control practices were assessed with questions about regular compliance with IPC precautions as well as through the use of three HCW-patient contact scenarios involving a patient who is diagnosed with a) respiratory tract infection (e.g. RSV) that is spread by droplets; b) active pulmonary tuberculosis, who has a productive cough; and c) varicella (chickenpox). Respondents were asked about the precautions they would take - such as wearing surgical mask, N95 respirator, face or eye shield, gloves, gown, or goggles - in each of these three scenarios. Additionally, for scenario (a), they were asked to provide a response in a situation when they are within 1 m (3 ft) of the patient with respiratory tract infection. Also, for scenario (c), they were asked to provide a response assuming they had immunity to varicella (e.g., via childhood infection). Quantitative responses were measured on a 1 to 10 scale. Charting practices regarding the accurate recording of number of daily patient-HCW interactions were also assessed.

## Results

Two thousand eight hundred thirteen staff completed paper questionnaires while 235 completed electronic surveys. Three thousand forty-eight HCW participated (38%), which exceeded our target participation rate by three-fold.

The distributions of survey participation by hospital and occupation are summarized in Table 2. Nurses were the occupational category with the highest aggregate response rate, although more administrative/support staff responded in Hospital A.

**Table 2:** Occupational response rates for each hospital surveyed

	Admin/Sup N (%)	Nurses *N* (%)	Other HCW *N* (%)	Physicians *N* (%)	Total Respondents^a^
Hospital A	753 (46.3)	591 (36.4)	173 (10.6)	108 (6.6)	1625
Hospital B	233 (28.7)	346 (42.7)	152 (18.7)	80 (9.9)	811
Hospital C	129 (22.2)	238 (40.9)	137 (23.6)	78 (13.4)	582
Total (%)	1115 (36.9)	1175 (38.9)	462 (15.3)	266 (8.8)	3018

^a^30 non-categorized responses were excluded from this table

The median age of respondents across all sites was 42 years, 81% were female, and most (75%) worked in a patient-care area. More than one third (37%) of physicians worked in other healthcare facilities in addition to the study hospital (Table 3).

**Table 3:** Summary of location data for each occupational category

	Provided location data (#)	Visited > 4 floors per week (%)	Visited only 1 floor per week (%)	Works in patient care area (%)	Also works in another healthcare facility (%)
Admin/Sup	1031	29.5	16.3	42	9
Nurses	1143	20.1	15.3	98	13
Other HCW	452	45.6	7.96	81	15
Physicians	254	47.2	10.6	92	37
All Categories	2880	29.9	14.1	75	14

Staff visited an average of 3.79, 3.69 and 3.88 floors in their respective healthcare facility per week, with a standard deviation of 2.63, 1.74 and 2.08. Physicians reported the highest number of locations visited per week, while nurses reported the lowest. The number of locations visited varied significantly depending on job category (Table 3). Results from Tukey post-hoc analyses showed nurses visited significantly fewer locations compared to physicians, “other HCW” and admin/support (*p* = 0.002, *p* = 0.001, *p* < 0.001, respectively).

Table 4 details the amount and type of contacts for each occupational group. Physicians reported the highest number of direct patient contacts (> 20 patients/day) but the lowest number of contacts with other HCWs, while nurses had the most extended (> 20 min) periods of direct patient contact. oHCWs had the most direct daily contact with other HCWs (Table 4).

**Table 4:** Number of contacts per occupational category

Occupational Category →	Admin/Sup	Nurses	Other HCW	Physicians %	Total %
Question ↓					
A1) Direct contact with patients per day	1019	1159	459	262	2899
A2) Direct contact with > 20 patients per day (% of A1)	261 (25.6%)	260 (22.4%)	106 (23.0%)	88 (33.6%)	715 (24.7%)
B1) Direct contact with any one patient per day	1022	1158	458	262	2900
B2) > 20 min of direct contact with any one patient per day (% of B1)	71 (6.9%)	781 (67.4%)	237 (51.7%)	77 (29.4%)	1166 (40.2%)
C1) Indirect contact with patients per day	985	1140	449	249	2823
C2) Indirect contact with > 20 patients per day (% of C1)	225 (22.8%)	274 (24.0%)	130 (29.0%)	60 (24.1%)	689 (24.4%)
D1) Direct contact with HCWs/day	1012	1140	445	243	2840
D2) Direct contact with > 20 HCWs/day (% of D1)	223 (22.0%)	253 (22.2%)	127 (28.5%)	34 (14.0%)	637 (22.4%)

Table 4 shows the number of contacts per occupational category. The first row in each pair corresponds to the number of respondents who answered questions labeled A1,. .., D1; the second row shows the percentage of these responses that satisfied the stated criteria (e.g., had direct contacts lasted more than 20 min).

### Contact network visualizations

**Fig. 1:**
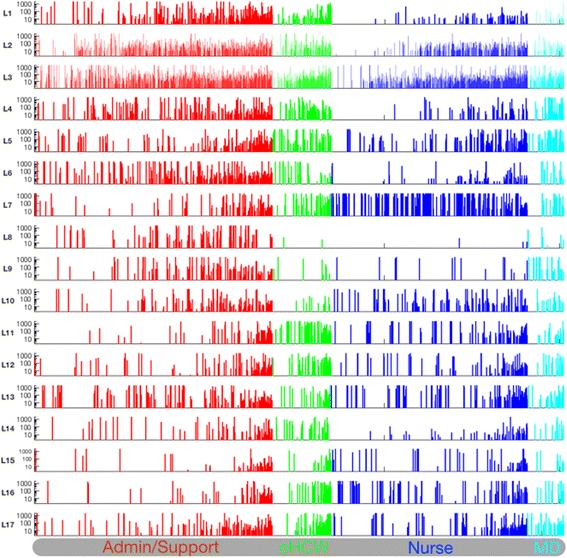
Detailed data corresponding to the time spent by HCWs on each floor of one study site during a typical week. Please see the main text for details. Different floors are labeled from L1 – L17. The bars are not sorted so that each HCW is represented on exactly same location on all 17 horizontal axes. Floor labels 1–17 correspond to floor levels L1 – L17 in Fig. 1, respectively

To provide additional insight into the aggregate statistics presented in Tables 3 and 4, Fig. 1 illustrates HCWs’ time spent on each floor at one of the participating study hospitals. Each bar chart (row) in this figure corresponds to a separate floor in that hospital (labeled L1 – L17). Along the horizontal axis, 1512 thin bars represent 679 administrative/support staff (red), 561 nurses (blue), 104 physicians (cyan) and 168 oHCW (green), who responded to the survey. The vertical axis represents time in logarithmic scale; each bar’s height reflects the time reported by that worker as having been spent on that floor. Thus, if a HCW reported spending a few-, up to 100 min on any single floor, the bar representing her/him can rise to the middle tick on the vertical axis; if hundreds of minutes, the bar may end in the middle segment of the y-axis; and finally, if few thousand minutes (up to a full work week), the bar may end on the upper segment of the y-axis. Also, in this figure, if a HCW spends time on more than one floor during the week, then they are represented by non-zero bars in the bar charts corresponding to those floors (and blank space in bar charts corresponding to other floors). There is a great variability in terms of the reported time spent, during a single, multiple, or routine visit(s), on each floor ranging from a few minutes to nearly a full work week (35 h/week = 2100 min/week).

**Fig. 2:**
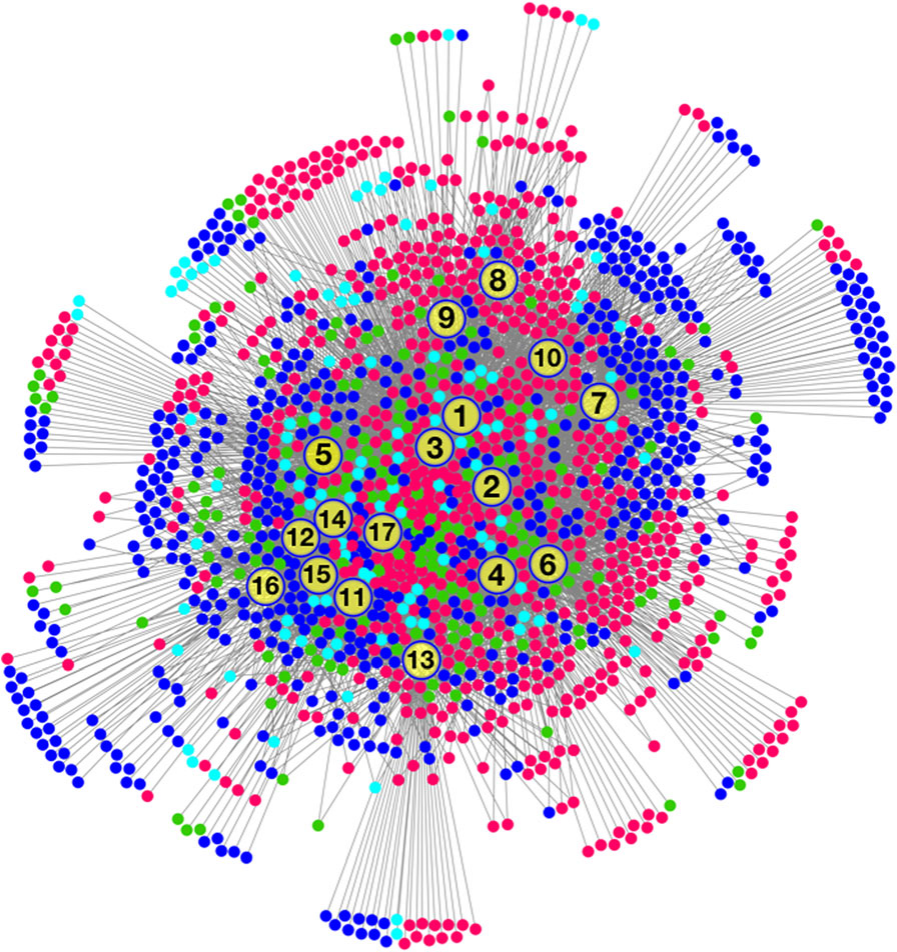
HCW-location bipartite network constructed from survey data collected from one of the participating hospitals. This network is weighted by duration of visits and differentiated by type of HCW (red: administrative/support staff, 679 nodes; blue: nurses, 561 nodes; cyan: physicians, 104 nodes; and green: oHCW, 168 nodes). Rather than organizing all floors (larger yellow nodes) on a straight line, the positions of these nodes are adjusted to allow for better visualization of clustering effect among other nodes

Based on data shown in Fig. 1, we generated a visualization of the ***bipartite network*** that captures HCW movement within a hospital setting (Fig. 2). A bipartite network shows the relationship between two distinct classes of nodes, in this case hospital floors and HCWs. Here the array of larger yellow nodes represents different floors in Hospital A, while all other nodes represent HCWs. An edge (black line) is drawn between a specific HCW and a location when the HCW reported visiting that location. HCW nodes are colored based on their occupational category.

The heterogeneity in the duration of time spent by a HCW in a spatial unit implies that the links connecting hospital floor and HCW do not have equal significance with respect to respiratory-borne infection transmission. For low- to moderately contagious infections, the probability of transmission among contacts in close-proximity is generally considered to be proportional to the duration of contact for each pair of individuals [22–24]. To account for the duration, each link should be weighted according to the length of time spent in a spatial unit; the longer the duration, the higher the weight.

Incorporating weighted edges in the network results in a gravity-centered network layout shown in Fig. 2, where edges with higher weights (“stronger” edges) and their associated nodes are concentrated near the core, while edges with lower weights (“weaker” edges), and their associated nodes are pushed outward to the periphery of the network.

The irregular density of edges in Fig. 2 reveals considerable heterogeneity in both the number and duration of contacts in the study hospitals. For infectious pathogens whose probability of transmission is proportional to the duration of contact (directly between individuals, or indirectly between a person and a spatial unit), this may have a significant impact on the transmission pathways within a healthcare setting. The likelihood of igniting a HAI outbreak, or being infected during such an outbreak, is higher for the nodes that are part of the central cluster than the ones belonging to the dendritic branches in the periphery of the network.

**Fig. 3:**
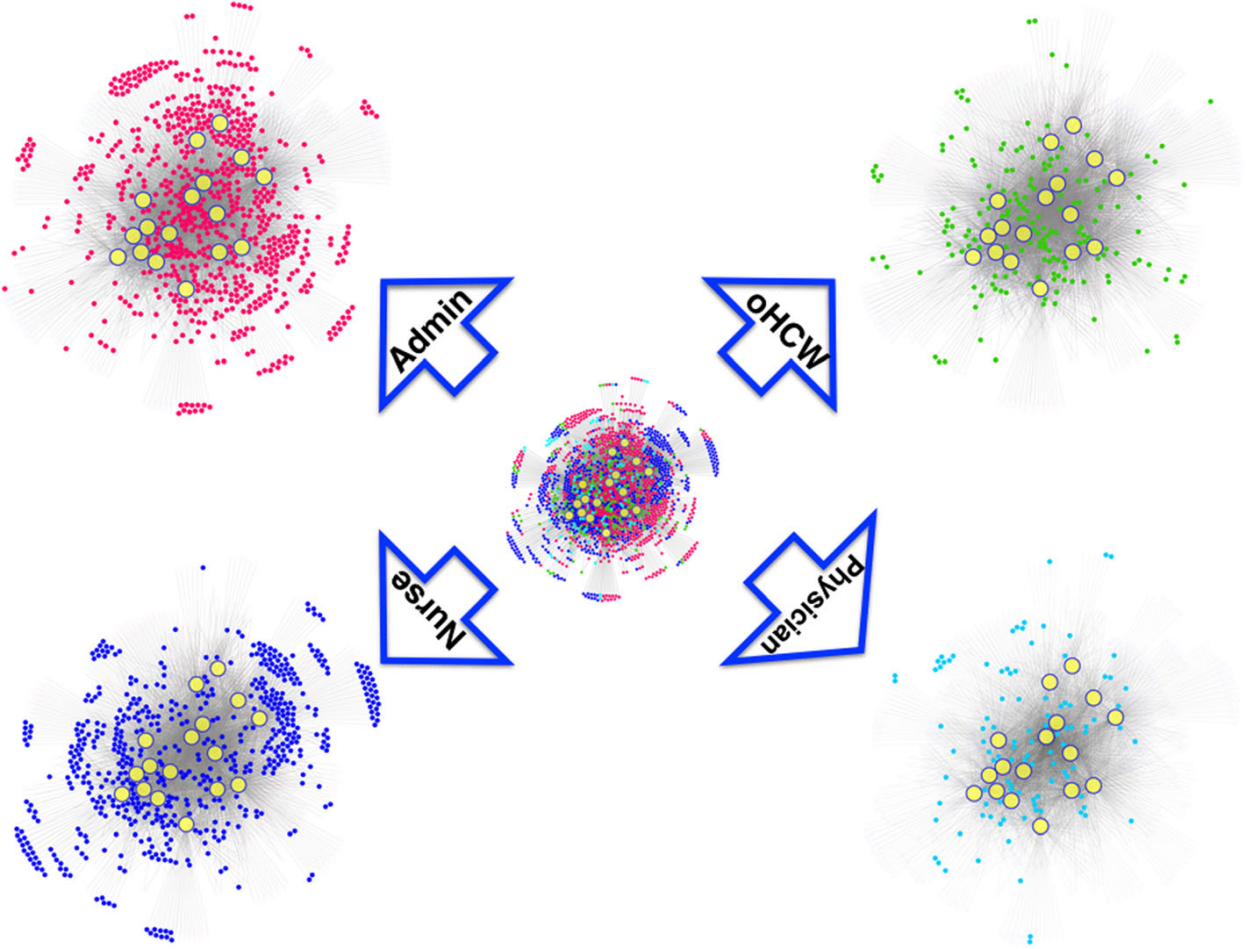
HCW-location bipartite network stratified by occupational category (red: administrative/support staff; blue: nurses; cyan: physicians; and green: oHCW)

Decomposing this network structure into its constituent occupational categories further exposes this heterogeneity. Fig. 3 shows the underlying weighted networks of the four occupational categories stratified around a central image that is a smaller replica of the full network (i.e., Fig. 2). While most nodes corresponding to participating Administration and Nurse categories occupy the peripheral branches of the weighted network, the majority of physicians are clustered in the centre (grey background area in all panels).

For location analyses, both the number of visits per week, and the mean hours spent, significantly differed by location type (Table 5). Public spaces had the most visits per week but the fewest mean hours spent (0.9 h). Inpatient settings had significantly more visits per week than outpatient settings.

**Table 5:** Summary of number of visits per week and hours spent per week corresponding to each hospital in patient care (PCA), non-patient care (Non PCA), and mixed (mPCA) areas

		Non PCA	mPCA	PCA
Hospital 1 1512 Respondents	Total number (and %) of floor visits per week	1277 (22.3%)	2725 (47.5%)	1734 (30.2%)
	Average time spent per floor (hrs/week)	6.5	7.5	13.0
Hospital 2 801 Respondents	Total number (and %) of floor visits per week	193 (6.5%)	1262 (42.7%)	1501 (50.8%)
	Average time spent per floor (hrs/week)	4.9	7.7	11.7
Hospital 3 568 Respondents	Total number (and %) of floor visits per week	464 (21.0%)	590 (26.7%)	1153 (52.2%)
	Average time spent per floor (hrs/week)	3.7	9.0	11.8

**Fig. 4:**
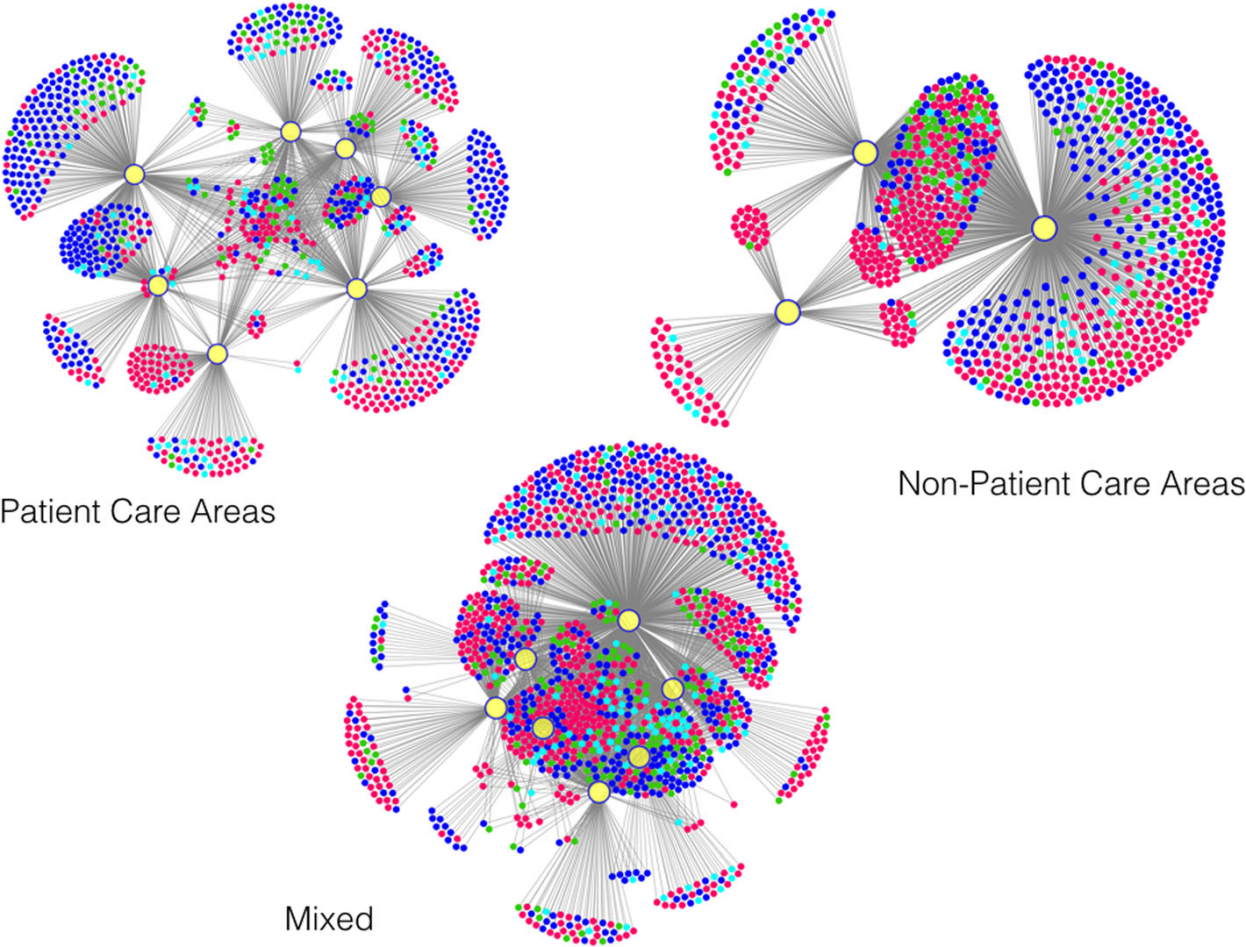
Three disjoint networks based on hospital floors’ classification as PCA, non-PCA, or mixed (red: administrative/support staff; blue: nurses; cyan: physicians; and green: oHCW)

The network in Fig. 2 can be divided into 3 disjoint networks based on hospital floors’ classification as PCA, non-PCA, or mixed (Fig. 4). The sub-network for PCA (top-left panel in Fig. 4) shows 3 different patterns: nodes (outer clusters) corresponding to HCW who visit only one floor; nodes (intermediate clusters) interact within two floors; and the remaining nodes (central core) representing individuals who visit several floors.

Comparatively speaking, floors with predominantly non-PCA areas (top right panel in Fig. 4) have higher between-floor traffic rate than PCA floors (top left panel). The highest between-floor HCW traffic occurs in mixed areas (lower panel in Fig. 4).

**Fig. 5:**
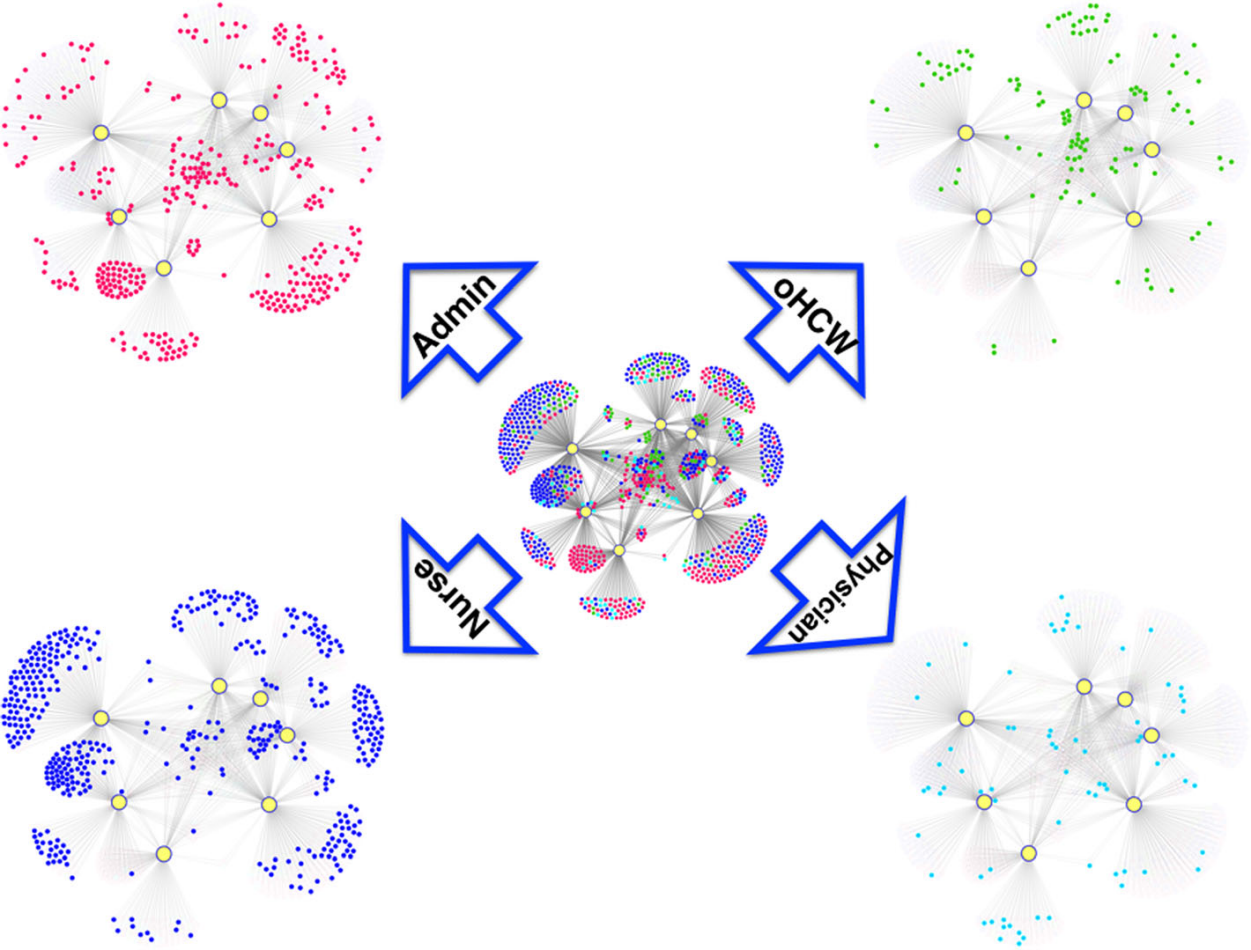
The sub-network corresponding to the PCA floors stratified by four occupational categories (red: administrative/support staff; blue: nurses; cyan: physicians; and green: oHCW)

Finally, as with Figs. 2 and 3, the sub-network corresponding to the PCA floors (top left panel in Fig. 4) may be stratified into the four occupational categories (Fig. 5). All occupational categories include nodes that report movement between multiple PCA floors (i.e., the most central clusters of nodes, Table 6). This movement may contribute to increasing the likelihood of an infectious transmission event within PCA floors.

**Table 6:** Summary of number of visits per week and hours spent per week for each category in patient care (PCA), non-patient care (Non PCA), and mixed (mPCA) areas

		Non PCA	mPCA	PCA
Admin/Supp (*N* = 1031)	Total number (and %) of floor visits per week	960 (30.2%)	1384 (43.6%)	831 (26.2%)
	Average time spent per floor (hrs/week)	6.3	8.0	8.4
Other HCW (*N* = 452)	Total number (and %) of floor visits per week	322 (15.9%)	791 (38.9%)	918 (45.2%)
	Average time spent per floor (hrs/week)	3.0	9.6	8.6
Nurses (*N* = 1144)	Total number (and %) of floor visits per week	522 (13.7%)	1577 (41.4%)	1709 (44.9%)
	Average time spent per floor (hrs/week)	1.2	7.1	16.9
Physicians (*N* = 254)	Total number (and %) of floor visits per week	130 (11.6%)	466 (41.6%)	525 (46.8%)
	Average time spent per floor (hrs/week)	1.4	8.0	11.1

### Infection prevention and control practices

Although respondents reported that they believed the majority of their HCW colleagues would comply with IPC guidelines (61.5% “mostly” comply, 31.5% “partially” comply), there was wide variability in reported use of personal protective equipment and only 81–87% expected compliance with handwashing after interacting with patients with communicable respiratory diseases (Table 7). Additionally, most respondents believed that patient charts would inaccurately report single or multiple HCW-patient interactions.

**Table 7:** HCW self-reported compliance with infection control guidelines and use of personal protective equipment (PPE)

How regularly do you think colleagues comply with infection control guidelines (*N* = 2857)	Mostly comply	1757 (61.5%)
	Partially comply	900 (31.5%)
	Poorly comply	200 (7%)
On average, how regularly direct contacts with patients recorded in patient’s chart (*N* = 2897)	Most often	1125 (38.8%)
	Sometimes	395 (13.6%)
	Not frequently	1226 (42.3%)
	N/A	151 (5.2%)
How regularly record multiple contacts with same patient in patient’s chart (*N* = 2803)	Most often	958 (34.2%)
	Sometimes	341 (12.2%)
	Not frequently	1300 (46.4%)
	N/A	204 (7.3%)
Wear surgical/procedure mask when caring for patient with. .. (*N* = 3002)	respiratory tract infection (RTI)	1414 (47.1%)
	TB	N/A
	Chickenpox	N/A
Wear N95 respirator (not fit tested) when caring for patient with. ..	RTI	171 (5.7%)
	TB	232 (7.7%)
	Chickenpox	75 (2.5%)
Wear N95 respirator (fit tested) when caring for patient with. . .	RTI	844 (28.1%)
	TB	1592 (53.0%)
	Chickenpox	345 (11.5%)
Wear face or eye shield when caring for patient with. . .	RTI	621 (20.7%)
	TB	1016 (33.8%)
	Chickenpox	N/A
Wear one pair of gloves when caring for patient with. . .	RTI	1757 (58.5%)
	TB	N/A
	Chickenpox	1618 (53.9%)
Wear two pairs of gloves when caring for patient with. . .	RTI	364 (12.1%)
	TB	N/A
	Chickenpox	273 (9.1%)
Wear goggles when caring for patient with. . .	RTI	352 (11.7%)
	TB	606 (20.2%)
	Chickenpox	N/A
Wear gown when caring for patient with. . .	RTI	1755 (58.5%)
	TB	N/A
	Chickenpox	1476 (49.2%)
Wash hands when caring for patient with. . .	RTI	2617 (87.2%)
	TB	2530 (84.3%)
	Chickenpox	2444 (81.4%)

## Discussion

The *CONNECT I* survey results presented here provide the most comprehensive picture of hospital-wide contact networks yet published. These insights provide evidence to support the development of novel network-based strategies for the prevention and control of HAI. Since the SARS outbreaks in 2003, there has been an emerging recognition of the complexity of hospital-based contact structures, and that this complexity varies by occupational type [25]. While prior studies have focused on individual hospital wards [18, 19, 26], patient-to-patient contact [15], or simulated/hypothetical patient-to-HCW contact [27], we report on actual self-reported patterns of movement and contact of over 3000 HCW in three Canadian urban tertiary care university affiliated hospitals. The resulting facility-specific networks identify occupational categories and specific locations within each unique setting that have high and low contact rates. Such data that can be utilized to inform targeted and efficient IPC strategies.

Contact and movement patterns of HCWs varied significantly by occupation. Although more nurses reported extended periods of direct patient contact, “other HCWs” (non-physician, non-nurse) had significantly more HCW contact per week than any other occupational category. In this paper, we aggregated HCW occupations into 4 main categories. We recognize HCW occupations such as respiratory therapists and personal care attendants may play a key role in spreading micro-organisms through physical contact, procedures such as intubation, or patient movement through the hospital. A higher resolution analysis of the survey data to address more refined questions may constitute the subject of future publications. The mobility of these occupations within a hospital may facilitate disease propagation compared to a more localized (within ward) movement, such as for nurses. Modeling the movement of these healthcare workers in the hospital setting may provide further insight into the propagation of diseases throughout the hospital.

We found that physicians, although mobile throughout the hospital, have a lower length of contact with other HCWs compared to any other occupational category, where a contact was defined as within 1 m of another individual for 2 min or more. This agrees with a study on one pediatric ward by Isella et al. [19], which found physicians to have the least number of contacts of the occupations surveyed and where a contact was defined as within 1.5 m for 20 s or more. In contrast, Polgreen et al. [17] found that nurses, resident physicians and fellows had the highest number of HCW contacts of the job categories observed, where a contact was defined as within 0.9 m, but had no minimum time component (i.e., duration of contact). More recently, Mastrandrea [26], studying a single infectious disease ward using radio frequency tracking devices, also found that physicians had the highest number of contacts with other health care workers, although this was within a total pool of only 22 HCWs. A study by Curtis et al. [16], used movement patterns from electronic medical records to suggest that resident physicians and nurses had the most frequent HCW contacts. While this conflicts with other findings, their definition of a contact differs significantly and did not include contacts in areas where electronic medical records fail to capture.

Despite their lower HCW contact rate in our study, physicians may still play a key role in infection-related events in the hospital. For example, significantly more physicians reported direct patient contacts of > 20 patients per day and were most likely to work in an additional but separate healthcare facility. This indicates that physicians may have a higher capacity to facilitate disease spread that propagates across wards and from hospital to hospital. Nurses reported the most extended contact with patients, and so may be at a higher risk of becoming infected by a patient. On the other hand, due to their more localized work space (typically a single ward), they may have a reduced role in the spreading of disease throughout the hospital population.

Location analyses showed that public spaces, including the cafeteria, lobby café, and coffee shops, were visited the most frequently per week but for a relatively shorter duration of time; this finding highlights a potential vulnerability of non-clinical spaces in healthcare facilities to promote infection spread for moderately- to highly transmissible pathogens. Given the vast overlap of HCWs, patients and the general public that may simultaneously visit these areas, disease spread could easily be facilitated between otherwise unconnected wards or units (or the community at large). Targeting these high-traffic areas with interventions such as hand-hygiene (washing stations or alcohol-based sanitizers), or mask distribution, or facilitating spatial separation may be effective in reaching a large and diverse subset of the hospital population.

Inpatient locations were found to have a greater number of visits per week compared to outpatient locations. Inpatients settings have patients with a higher acuity of illness, and therefore a greater diversity of HCWs may be in contact with the patient. This suggests that there is an increased risk of disease spread in inpatient settings compared to outpatient settings.

Variable compliance in implementing and incorrect application of IPC precautions combined with the non-intuitively diverse structure of HCW contact patterns shown above, may lead to complex infection transmission dynamics pathways. Accounting for this complexity will require the use of quantitative complexity science techniques that go beyond basic statistical description of survey data.

As with any paper-based questionnaire, one of the limitations of this study was that the responses relied on an individual’s recollection of movement throughout the hospital. To minimize the impact of this limitation, the respondents were given the choices of providing their contact history data based on a “typical week” of work, or “last week” of work, or “the last full week worked”. After the paper questionnaires were distributed within participating hospitals, drop boxes were provided for several weeks at study sites to collect responses. We assume among those who selected “last week”, some might have had a chance to assess their responses in “real time”, while others relied on their immediate-past, or past memories (typical week).

In the questionnaire, we clarified direct contacts as those that occur “within 1 meter/3 feet”, while indirect contacts are those that occur “within the same room but not closer than 1 meter/3 feet”. Although based on these definitions, these two types of contact are mutually exclusive, it might have been difficult for respondents to strictly apply these definitions when recalling (immediate) past events. It is worth noting that in addition to the duration of contact, the type and intensity of contact are among factors to be considered, as physical contact might play an important role for some HCAIs [22, 23]. In this paper, our goal was not to construct direct contact networks between HCWs (i.e., all nodes in the network representing HCWs); rather, we presented co-location networks (i.e., bipartite HCW-location networks) derived from survey data. As such, we used each hospital floor as a single node in networks for ease of presentation. To establish formal inter-HCW contact networks, for outbreak and transmission dynamics analysis, future studies will utilize *CONNECT I*’s more refined data corresponding to smaller spatial units (please see Additional file 1) than floor-aggregated data.

## Conclusion

The network structures presented in this paper reveal a high degree of heterogeneity across HCW occupations and their roles on different wards/floors. These intricacies combined with heterogeneity in implementing IPC measures imply that designing policy requires employing network-based quantitative tools beyond that of basic aggregate statistics. These tools provide greater options and flexibility based on specific contact patterns that facilitate communicable disease transmission within hospital settings. Future research based on heterogeneity of movement patterns across the 3 hospitals will allow further tailoring of interventions for setting-specific control strategies.

The *CONNECT I* study provides insight into the movement and contact patterns of healthcare workers in the general hospital setting. These results can inform modeling initiatives to more accurately simulate the spread of HAI, and to optimize control strategies.

## Additional files

Additional file 1: CONNECT I Questionnaire. Description of data: A generic version of site-specific questionnaire used to collect data from participating hospitals. (PDF 141 kb)

## Abbreviations

ANOVA: Analysis of variance
CIHR: Canadian Institutes of Health Research
HAI: Healthcare-associated infection
HCW: Health care worker
IPC: Infection prevention and control
MERS: Middle East respiratory syndrome
mPCA: Mixed patient-care area
Non-PCA: Non-patient-care area
oHCW: Other HCWs (non-physician, non-nurse)
PCA: Patient-care area
RFID: Radio frequency identification
RSV: Respiratory syncytial virus
SARS: Severe acute respiratory syndrome

## References

[1] Zoutman DE, Ford BD, Bryce E, Gourdeau M, Hébert G, Henderson E (2003). The state of infection surveillance and control in Canadian acute care hospitals. Am J Infect Control.

[2] Umscheid CA, Mitchell MD, Doshi JA, Agarwal R, Williams K, Brennan PJ (2015). Estimating the proportion of healthcare-associated infections that are reasonably preventable and the related mortality and costs. Infect Control Hosp Epidemiol.

[3] Marschang S, Bernardo G (2015). Prevention and control of healthcare-associated infection in Europe: a review of patients' perspectives and existing differences. J Hosp Infect.

[4] Public Health Agency of Canada (2012). Routine practices and additional precautions for preventing the transmission of infection in healthcare settings.

[5] CDC (1998). SPECIAL ARTICLE guideline for infection control in health care personnel, 1998.

[6] Pourbohloul B, Meyers LA, Skowronski DM, Krajden M, Patrick DM, Brunham RC (2005). Modeling control strategies of respiratory pathogens. Emerg Infect Dis.

[7] Meyers LA, Pourbohloul B, Newman MEJ, Skowronski DM, Brunham RC (2005). Network theory and SARS: predicting outbreak diversity. J Theor Biol.

[8] Mossong J, Hens N, Jit M, Beutels P, Auranen K, Mikolajczyk R, Riley S (2008). Social contacts and mixing patterns relevant to the spread of infectious diseases.

[9] Zagheni E, Billari FC, Manfredi P, Melegaro A, Mossong J, Edmunds WJ (2008). Using time-use data to parameterize models for the spread of close-contact infectious diseases. Am J Epidemiol.

[10] Smieszek T, Barclay VC, Seeni I, Rainey JJ, Gao H, Uzicanin A (2014). How should social mixing be measured: comparing web-based survey and sensor-based methods. BMC Infect Dis.

[11] Smieszek T, Castell S, Barrat A, Cattuto C, White PJ, Krause G. Contact diaries versus wearable proximity sensors in measuring contact patterns at a conference: method comparison and participants’ attitudes. BMC Infect Dis. 2016. p. 1–14. 10.1186/s12879-016-1676-y. 10.1186/s12879-016-1676-y PMC4957345 27449511

[12] Rossana Mastrandrea JFAB (2015). Contact patterns in a high school: a comparison between data collected using wearable sensors, contact diaries and friendship surveys.

[13] Bernard H, Fischer R, Mikolajczyk RT, Kretzschmar M, Wildner M (2009). Nurses’ contacts and potential for infectious disease transmission. Emerg Infect Dis.

[14] Ueno T, Masuda N (2008). Controlling nosocomial infection based on structure of hospital social networks. J Theor Biol.

[15] Cusumano-Towner M, Li DY, Tuo S, Krishnan G, Maslove DM (2013). A social network of hospital acquired infection built from electronic medical record data. J Am Med Inform Assoc.

[16] Curtis DE, Hlady CS, Kanade G, Pemmaraju SV, Polgreen PM, Segre AM, Colizza V (2013). Healthcare worker contact networks and the prevention of hospital-acquired infections.

[17] Polgreen PM, Tassier TL, Pemmaraju SV, Segre AM (2015). Prioritizing healthcare worker vaccinations on the basis of social network analysis. Infect Control Hosp Epidemiol.

[18] Hornbeck T, Naylor D, Segre AM, Thomas G, Herman T, Polgreen PM (2012). Using sensor networks to study the effect of peripatetic healthcare workers on the spread of hospital-associated infections. J Infect Dis.

[19] Isella L, Romano M, Barrat A, Cattuto C, Colizza V, Van den Broeck W, Cowling B (2011). Close encounters in a pediatric Ward: measuring face-to-face proximity and mixing patterns with wearable sensors.

[20] Vanhems P, Barrat A, Cattuto C, Pinton J-F, Khanafer N, Régis C, Viboud C (2013). Estimating potential infection transmission routes in hospital wards using wearable proximity sensors.

[21] Siegel JD, Rhinehart E, Jackson M, Chiarello L (2007). 2007 guideline for isolation precautions: preventing transmission of infectious agents in health care settings. Am J Infect Control.

[22] Smieszek T (2009). A mechanistic model of infection: why duration and intensity of contacts should be included in models of disease spread. Theor Biol Med Model.

[23] De Cao E, Zagheni E, Manfredi P, Melegaro A (2014). The relative importance of frequency of contacts and duration of exposure for the spread of directly transmitted infections. Biostatistics.

[24] Haas CN, Rose JB (2014). Gerba CP.

[25] Raboud J, Shigayeva A, McGeer A, Bontovics E, Chapman M, Gravel D, Montgomery JM (2010). Risk factors for SARS transmission from patients requiring intubation: a multicentre investigation in Toronto, Canada.

[26] Mastrandrea R, Soto-Aladro A, Brouqui P, Barrat A. Enhancing the evaluation of pathogen transmission risk in a hospital by merging hand-hygiene compliance and contact data: a proof-of-concept study. BMC Research Notes. BioMed Central. 2015. p. 1–11. 10.1186/s13104-015-1409-0. 10.1186/s13104-015-1409-0 PMC4566487 26358118

[27] Raboud J, Saskin R, Simor A, Loeb M, Green K, Low DE (2016). Modeling transmission of methicillin-resistant staphylococcus aureus among patients admitted to a hospital. Infect Control Hosp Epidemiol.

