# Supplementary material for: Contact among healthcare workers in the hospital setting: developing the evidence base for innovative approaches to infection control
Source: BMC Infect Dis. 2018 Apr 17;18:184. doi: 10.1186/s12879-018-3093-x (PMC5905140; doi:10.1186/s12879-018-3093-x)
Supplement: Supplementary file 1 — CONNECT I Questionnaire. Description of data: A generic version of site-specific questionnaire used to collect data from participating hospitals. (PDF 141 kb) [file 12879_2018_3093_MOESM1_ESM.pdf]

Supplementary Material

Contact among healthcare workers in the hospital setting: Developing the evidence base for innovative approaches to infection control

Krista M. English, Joanne M. Langley, Allison McGeer, Nathaniel Hupert, Raymond Tellier, Bonnie Henry, Scott A. Halperin, Lynn Johnston, Babak Pourbohloul

As stated in the manuscript, the *CONNECT I* study was based on site-specific surveys for three urban university-affiliated tertiary care Canadian hospitals. The data collection instruments were hard-copy paper booklets with information packages and 1 online survey. The paper surveys were to be completed by HCWs and returned anonymously to a centrally-located drop box. Below, is a generic copy of the questionnaire. All questions remained the same in the questionnaires distributed in different hospital, with the exception of Question #11. This question pertains to the time spent by a HCW in different locations in a hospital. Based on each hospital's architectural maps and floor plans, this question includes detailed information about various units located on each floor. In the following generic questionnaire, we assume an X-storey hospital, with K units on the 1st floor, L units on the 2nd floor, . . . and M units on the Xth floor. In our study, 251, 122, and 97 space units were included in Question # 11, depending on the participating hospital. As such, the content of this question may take few pages on a paper questionnaire.

Demographics

1. Sex ☐ M ☐ F

2. Year of Birth: ________________

3. Primary ward or location within which you work:
____ or ☐ Rotating

4. Occupation:
☐ Staff Physician, specialty: _____________________________________
☐ Nurse
☐ Phlebotomist/IV nurse
☐ Nursing Aide
☐ LPN
☐ Patient Attendant/Sitter
☐ Diagnostic Imaging Technologist
☐ Pharmacist
☐ Undergraduate Medical Trainees
☐ Postgraduate Medical Trainees
☐ Other student (excluding medical students), discipline: ______________
☐ Ward Clerk
☐ Physiotherapist/Occupational Therapist

☐ Respiratory Therapist
☐ Housekeeping
☐ Social Worker
☐ Porter
☐ Central Sterile Supply
☐ Other, specify: ____

5. Do you also work in any other health care settings such as another hospital, clinic, nursing home, etc.?
☐ No	If yes: ☐ Employed	☐ Volunteer

6. Are you employed in any other non-health care settings?
☐ No	☐ Yes

If yes, please specify: ____

7. Number in household (including self): ___________________________

8. Ages of others in household (if any): ____________________________

9. Type of dwelling: ☐ Detached House	☐ Townhouse
☐ Condominium/Apartment, please specify number of units:
☐ <50	☐ 50-99	☐ 100-199	☐ 200+ units
☐ Other, specify: ____

Occupational History

10. On average, how many hours do you work at this hospital per week?
____

The following questions ask you about your activity in a **typical week**.

In order to understand how infections spread through a hospital setting, we need to know your physical movement in a **typical week**. Think of it as tracking your every move. That is the best way for us to see how infections spread. As you complete the following questions, think of all the locations you have worked or visited during the week. We are asking that you list the locations and how long you were at the location. Remember to include things like going to Xray with a patient, dropping off samples to the lab, going to the cafeteria or Tim Horton's.

As you complete these questions, imagine that you are watching a video of your movement and you are writing down all that you see. The more you can tell us, the better we may understand how infections can be spread. If you cannot remember a typical week then try to remember the **last full week** you worked.

11. Please check all the areas you worked in during a typical week and enter the hours and/or minutes in spaces provided.

<u>HOSPITAL NAME 1st Floor</u>

Unit Name		Hours	Minutes	Unit Name		Hours	Minutes
LOCATION NAME 1	☐	_____	_____	LOCATION NAME 2	☐	_____	_____
LOCATION NAME 3	☐	_____	_____	LOCATION NAME 4	☐	_____	_____
LOCATION NAME 5	☐	_____	_____	LOCATION NAME 6	☐	_____	_____
• • •	•	•	•	• • •	•	•	•
• • •	•	•	•	• • •	•	•	•
• • •	•	•	•	• • •	•	•	•
• • •	•	•	•	LOCATION NAME **K**	☐	_____	_____

<u>HOSPITAL NAME 2nd Floor</u>

Unit Name		Hours	Minutes	Unit Name		Hours	Minutes
LOCATION NAME 1	☐	_____	_____	LOCATION NAME 2	☐	_____	_____
LOCATION NAME 3	☐	_____	_____	LOCATION NAME 4	☐	_____	_____
LOCATION NAME 5	☐	_____	_____	LOCATION NAME 6	☐	_____	_____
• • •	•	•	•	• • •	•	•	•
• • •	•	•	•	• • •	•	•	•
• • •	•	•	•	• • •	•	•	•
• • •	•	•	•	LOCATION NAME **L**	☐	_____	_____

<u>HOSPITAL NAME 3rd Floor</u>

•
•
•

•. . . . .
•
•

•. . . . .
•
•

<u>HOSPITAL NAME Xth Floor</u>

Unit Name		Hours	Minutes	Unit Name		Hours	Minutes
LOCATION NAME 1	☐	_____	_____	LOCATION NAME 2	☐	_____	_____
LOCATION NAME 3	☐	_____	_____	LOCATION NAME 4	☐	_____	_____
LOCATION NAME 5	☐	_____	_____	LOCATION NAME 6	☐	_____	_____
• • •	•	•	•	• • •	•	•	•
• • •	•	•	•	• • •	•	•	•
• • •	•	•	•	• • •	•	•	•
• • •	•	•	•	LOCATION NAME **M**	☐	_____	_____

12. I answered the above questions based on:
☐ a typical week, or ☐last week, or ☐the last full week worked

The following questions ask you about your activity on a **typical day**. If you cannot remember a typical day, then try to remember the **last full day** you worked.

13. On a typical day, with how many patients are you in **direct contact** (within 1 meter/3 feet)?
☐ <5 ☐ 5-9 ☐ 10–19 ☐ 20-29 ☐ 30-39 ☐ 40-49 ☐ 50+ patients

14. **On average**, how many minutes do you spend in **direct contact** with any one patient per day (within 1 meter/ 3 feet)?
☐ < 5 ☐ 5-10 ☐ 11-20 ☐ 20+ minutes

15. On average, with how many patients do you have **indirect contact** (same room but not closer than 1 meter/3 feet) per day?
☐ 1–5 ☐ 6-10 ☐ 11–15 ☐ 16-20 ☐ 21-25 ☐ 26-30 ☐ 30+ patients

16. On average, with how many health care co-workers (HCW) do you have **direct contact per day** (within 1 meter for at least two minutes)?
☐ 1–5 ☐ 6-10 ☐ 11–15 ☐ 16-20 ☐ 21-25 ☐ 26-30 ☐ 30+ HCWs

17. I answered the above questions based on:
☐a typical day, or ☐yesterday, or ☐the last full day worked

18. In your experience, how **regularly** do your colleagues comply with disease infection control guidelines? Please circle your response, rated from 0 (full non-compliance) and 10 (full compliance).

**Non-compliance**										**Compliance**
0	1	2	3	4	5	6	7	8	9	10

19. Sometimes, direct contact with a patient (within 1 meter) is a patient care activity that should be documented in the health record and sometimes not. On average, how **regularly** do you record contacts with a patient in the patient's health record?
Please circle your response, rated from 0 (never record) and 10 (always record).

**Never**										**Always**
0	1	2	3	4	5	6	7	8	9	10

20. Usually, you have more than one contact with a patient on a shift. If you have more than one direct contact with the same patient on any given day, how **regularly** would you record your name in the patient's chart?
Please circle your response, rated from 0 (never record) and 10 (always record).

Never										Always
0	1	2	3	4	5	6	7	8	9	10

21. Imagine a patient that is diagnosed with a **respiratory tract infection** (e.g. influenza, RSV, adenovirus) that is spread by droplets. Please check all precautions you would take when you are **within 1 metre (3 feet)** of this patient:

☐ surgical/procedure mask	☐ N95 mask (not fit tested)	☐ N95 mask (fit-tested)
☐ face or eye shield	☐ two pairs of gloves	☐ goggles
☐ single pair of gloves	☐ gown	☐ hand washing

22. How **regularly** do you comply with recommended infection prevention measures for patients with **respiratory tract infections** noted above?
Please circle your response, rated from 0 (never) to 10 (always).

Never										Always
0	1	2	3	4	5	6	7	8	9	10

23. Imagine a patient that is diagnosed with active **pulmonary tuberculosis** and who is coughing up mucus. Please check all precautions you would take when you are in the same room with this patient:

☐ surgical/procedure mask	☐ N95 mask (not fit tested)	☐ N95 mask (fit-tested)
☐ face or eye shield	☐ two pairs of gloves	☐ goggles
☐ single pair of gloves	☐ gown	☐ hand washing

24. How **regularly** are you able to comply with recommended infection prevention measures for patients with active **pulmonary tuberculosis** who are coughing up mucus?
Please circle your response, rated from 0 (never) and 1 (always).

Never										Always
0	1	2	3	4	5	6	7	8	9	10

25. Imagine a patient that is diagnosed with **chickenpox** and you have had the infection in childhood. Please check all precautions you would take when you are in the same room with this patient:

☐ surgical/procedure mask	☐ N95 mask (not fit tested)	☐ N95 mask (fit-tested)
☐ face or eye shield	☐ two pairs of gloves	☐ goggles
☐ single pair of gloves	☐ gown	☐ hand washing

26. How **regularly** are you able to comply with recommended infection prevention measures for patients with **chickenpox**? Please circle your response, rated from 0 (never) to 10 (always).

**Never**										**Always**
0	1	2	3	4	5	6	7	8	9	10

27. Do you get a yearly influenza vaccine?
☐ No (never) ☐ Yes, every year ☐ Yes, but not every year

28. On average, how many times per year do you come down with a cough or cold illness? ____

Thank you for completing this questionnaire. Please return the questionnaire to the study center using the self-addressed envelope.
